# Methane Emissions, Performance and Carcass Characteristics of Different Lines of Beef Steers Reared on Pasture and Finished in Feedlot

**DOI:** 10.3390/ani10020303

**Published:** 2020-02-13

**Authors:** Paulo Méo-Filho, Alexandre Berndt, Cintia R. Marcondes, André F. Pedroso, Leandro S. Sakamoto, Daniella F. V. Boas, Paulo H. M. Rodrigues, M. Jordana Rivero, Ives C. S. Bueno

**Affiliations:** 1 Faculty of Animal Science and Food Engineering, University of São Paulo, 225 Duque de Caxias Norte Ave, Pirassununga, São Paulo 13635-900, Brazil; 2 Embrapa Southeast Livestock, Rodovia Washington Luiz, km 234, São Carlos, São Paulo 13560-970, Brazil; 3 Faculty of Veterinary Medicine and Animal Science, University of São Paulo, Duque de Caxias North Ave, Pirassununga, São Paulo 13635-900, Brazil; 4 Rothamsted Research, North Wyke, Okehampton, Devon EX20 2SB, UK

**Keywords:** beef cattle, genetics, greenhouse gases, growth performance, nutrition

## Abstract

**Simple Summary:**

The livestock sector is a significant contributor to global greenhouse gas emissions, with cattle representing 62% of CO_2_ emissions. Genetic selection is a tool that has the potential to reduce emissions from rumen fermentation and can be adopted in grazing and feedlot systems. Therefore, the purpose of this study was to evaluate steers from a new line of the Canchim breed (5/8 Charolais, 3/8 Zebu) derived by genetic selection improvement, and formed and maintained as a closed herd since 1998, in regards to performance, carcass dressing, and enteric methane emissions, when compared with the old line formed and maintained as a closed herd since 1960. Animals from the new line of the Canchim breed should be used for rearing on pasture to obtain greater liveweight gain. However, genetic improvement in Canchim breed does not guarantee animals with lower methane emissions under grazing conditions. For the finishing feedlot phase, increased liveweight gain, improved feed conversion, and a better carcass dressing percentage are obtained using animals from the new line of Canchim breed. The new line also leads to higher daily feed intake and higher daily liveweight gain, but with the same methane emissions per kg of animal liveweight than the other lines.

**Abstract:**

The present study aimed to investigate whether different lines of a composite breed (5/8 Charolais × 3/8 Zebu), formed at different times, and genetically improved, would result in differences in animal performance, enteric methane emissions, and carcass traits. Forty-six Canchim steers (15 months, 280 ± 33 kg liveweight) from three different lines were used: old, new, and their cross. These three breed lines were considered the treatments (arranged in four randomized blocks based on initial liveweight) and were evaluated under grazing and feedlot conditions in relation to the performance and emission of enteric methane. During the grazing period, the new line was found to be superior to the old only in relation to the average daily liveweight gain (0.692 vs. 0.547 kg/day), and with no differences in relation to the cross line (0.692 vs. 0.620). In the feedlot finishing phase, only the average daily liveweight gain was significantly higher in the new line compared to the cross and old line (1.44 vs. 1.32 and 1.23 kg/day). The new and cross lines demonstrated higher dry matter intake when compared to the old line (10.25 and 10.42 vs. 9.11 kg/day), with the crossline animals demonstrating the best feed conversion. The new line showed higher enteric methane emissions compared to the old line (178 vs. 156 g/day). The line had an effect on the carcass dressing of the animals, with greater fat thickness in carcasses from the new and cross lines than the old line (4.4 and 3.8 vs. 3.2 mm). Canchim cattle selected for improved productive performance characteristics does not guarantee animals with lower methane emissions under grazing conditions; while in feedlots, can lead to increased daily feed consumption, and hence, to higher emissions of methane.

## 1. Introduction

Livestock farming is a fundamental activity for maintaining the current society. By 2050, the world’s population is expected to reach 9.1 billion people. As a result, agricultural activities must grow accordingly in order to sustainably meet this growing food demand [1]. According to Oliveira Silva et al. [2], ruminant animals are the largest cause of agricultural externalities in terms of land use and emissions of greenhouse gas (GHG), such as methane (CH_4_). In 2010, agriculture accounted for 5.5 gigatonnes of carbon dioxide equivalent (GtCO_2_eq) of global emissions, with 2.1 GtCO_2_eq of this total resulting from of enteric fermentation by these animals [3].

The approach adopted by the Intergovernmental Panel on Climate Change (IPCC) for GHG inventories, while adequate, does not take into account the mitigating options that exist within each activity. Cattle reared on pasture, for example, have significantly lower emissions when we take into account atmospheric carbon dioxide (CO_2_) that is absorbed by grasses and added to the stock of carbon (C) in the soil [4]. Considering that approximately 3.7 billion hectares of the earth’s surface are occupied by pasture, the potential for sequestration varies between 0.01 and 0.3 GtC/year [5]. Almeida and Medeiros [6] suggest the use of strategic feedlots for a short period during the finishing of beef cattle as an alternative for mitigation of GHG emissions. If the animal reaches slaughter weight earlier, its life cycle will be shorter, and consequently, it will emit less GHG. A similar effect occurs when more efficient animals are used [7].

The hypothesis of the present study was: Canchim breed cattle genetically improved for performance traits, even within lines of the same breed, will result in differences in animal performance, enteric methane emissions, and carcass traits. Therefore, the purpose of this study was to evaluate steers from different lines of the Canchim breed (5/8 Charolais, 3/8 Zebu) resulting from genetic selection programs, in regards to performance, carcass dressing, and enteric CH_4_ emissions.

## 2. Materials and Methods

### 2.1. Experimental Design and Treatments

The experiment took place between December 2014 and September 2015 at the Brazilian Agricultural Research Corporation (Embrapa Southeast Livestock, São Carlos, São Paulo). The trial was approved by the local committee for Ethics in Animal Experimentation (CEUA) (protocol number 03/2014).

Forty-Six Canchim steers (composite breed of 5/8 Charolais and 3/8 Zebu) from three distinct lines were used: old (n = 13), new (n = 20) and cross (n = 13, a result from the crossing of animals from the old and new lines). These three breed lines were considered the treatments. According to recommendations by Morris [8], within the breed lines, animals were arranged in four randomized blocks based on initial liveweight. Four pasture areas and four pens in feedlot were used to receive one block of each treatment (breed lines). Each area or pen comprised one block with the three treatments. The final arrangement is presented in Table 1.

### 2.2. Description of the Canchim Breed Lines

The Canchim breed was developed by the Brazilian Ministry of Agriculture at the then São Carlos Experimental Station (now Embrapa Southeast Livestock), from the 1940s, through the alternate crossing between Charolais and Zebu (Guzera, Indubrasil, and Nelore breeds). The overall objective was to take advantage of the favorable effects of heterosis (hybrid vigor), to complement the desirable characteristics of Charolais (high growth rate and good meat quality), and Zebu (adaptation to the tropical conditions), and to facilitate the introduction of Charolais genes into Brazilian beef production systems through the use of the Canchim breed [9]. The old line has been kept at Embrapa Southeast Livestock as a closed herd since 1953. It was originally formed from 53 Charolais bulls imported from France between 1922 and 1950 that were mated with Zebu females [10,11]. The breed’s genetic basis was limited to these resources until the early 1990s, when, after approval by the Ministry of Agriculture, Livestock and Supply, the process of formation of new lines was approved at the request of the Brazilian Association of Canchim Breeders. The development of the new line began at that time, when the genetic base of the breed began to enlarge with the use of Charolais bulls imported from different countries, and from breeding programs with proven genetic merit, and at that time, exclusively mated with Nellore females. After the establishment of the new line, it has been kept closed since 1998 [12]. Animals from the cross line began to be bred from 1998 to 2010 [13], when the group was closed. The aim was to study the effect of hybrid vigor obtained from the crossing of the old and new line.

From their formations, breeding animals of the respective lines were mated based on Expected Differences in Progeny (EPDs), provided by the Geneplus-Embrapa breeding program, where the following selection traits were used: birth weight, scrotal circumference, coat scores, belly button, cold shape, weaning liveweight, yearling liveweight, and age at first calving [10]. Regarding the relative selection pressure, the “Geneplus” Genetic Qualification Index (GQI) for the Canchim breed is: 0.15 BW + 0.20 MA+ 0.30 YL + 0.20 YCC + 0.15 YSC (where BW is birth weight; MA is maternal ability; YL is yearling liveweight; YCC is yearling carcass conformation; YSC is yearling scrotal circumference). In the experimental herd of Embrapa Southeast Livestock, since 2013, EPDs for BW, belly button and pelage are being controlled (values close to zero) and EPDs for YL, YCC, and YSC with greater emphasis on the use of animals above the average EPD value. Since 2013, through new experiments, unconventional criteria were studied to provide future production systems with heat tolerant animals, with good resistance to parasites, early maturing, and with better carcass finishing. Animals from different lines have been studied at Embrapa Southeast Livestock since 2011 [13], with the objective of comparing the productive characteristics of the three lines, as closed experimental herds.

### 2.3. Pasture Characteristics and Measurements

The animals within their respective lines were ranked according to their initial liveweight (to avoid competition), so that the heaviest animals of each line were placed with the heaviest of the other two lines, the intermediate with the intermediate and the lightest with the lightest, respectively, being then allocated to one of the four pasture areas (considered as a block) by drawn. Each area of pasture was composed of four modules with 12 strips each, following a variable rotational grazing method. Drinking water and a trough for mineral supplementation was always available. The pasture system was composed of *Panicum maximum* L. (cv. Tanzania), which received nitrogen fertilization after the animals were moved on from each strip, totaling 400 kg of N/ha per year. 

The grazing period adopted was three days per strip with a rest period of 33 days, totaling 36 days per cycle. The pasture rearing period (165 days) permitted the observation of 4 full grazing cycles (4 × 36 d) plus 21 grazing days on the fifth grazing cycle (the last five strips were not grazed in this last grazing cycle). The stocking rate was adjusted following the put-and-take technique [14], to ensure a similar forage offer between the modules. To monitor liveweight (LW) gain, the animals were weighed after a fasting period of 16 h, at the beginning and at the end of the grazing phase, and without fasting every 36 days within this interval. 

Forage mass production was determined directly by the quadrat method, where the frame had a size of 1.0 m^2^, and a random choice of collection points (9 points per paddock). The material collected above the residue (40 cm) recommended height, according Barbosa et al. [15], was weighed, and a sub-sample taken to determine the dry matter was dried in an oven at 65 °C for 72 h. The mean forage yield during the five cycles evaluated was 2550 ± 648 kg DM/ha.

### 2.4. Animal Characteristics and Measurements

The animals from the experimental Canchim herd were reared at Embrapa Southeast Livestock, born between August and November 2013. At the start of the experiment, their LW was 280 ± 33 kg and were 15 ± 3 months of age, with 13 animals from the old line, 20 from the new line, and 13 from the cross line.

During the pasture rearing period, dry matter intake (DMI) was estimated individually using the prediction model proposed by Azevêdo et al. [16], considering the determined average LW (ALW) and average daily gain (ADG), where ALW is the average of the initial and final LWs during the pasture phase (used for calculating the ADG):

(1) $$\mathrm{DMI}\;=\;-2.6098\;+\;0.08844\mathrm{AL}W^{0.75}\;+\;4.4672\mathrm{ADG}\;-\;1.3579{\mathrm{ADG}}^{2}$$

This prediction model was adopted considering that it has been proposed based on a meta-analysis of 561 results obtained in 27 studies (thesis and dissertations) published by the Federal University of Viçosa and the University of São Paulo and is known as: “BR-CORTE” [17]. Although prediction models by the National Research Council (NRC) [18,19] can be applied in Brazilian studies, those models have been developed using mostly *Bos taurus* cattle data and, besides that, the database included results obtained from animals that had received anabolic steroids prohibited in Brazil since 1961. Studies that evaluated NRC [19] prediction models concluded that the equations proposed to predict DMI were not totally adequate and the development of new and more precise equations was suggested [20]. Valadares Filho et al. [21] also reported a lack of fit in models proposed by the NRC [18,19], when predicting DMI by beef cattle in tropical conditions. Ribeiro et al. [22] evaluated the prediction of DMI by genetic groups of Zebu cattle using models by the NRC [19], the Cornell Net Carbohydrate and Protein System - CNCPS 5.0 [23] and BR-CORTE [17], concluding that the Brazilian model [17] (BR-CORTE, 2006) was more accurate for beef cattle in tropical conditions.

### 2.5. Methane Emissions Sampling at Grazing

Emissions of enteric methane were measured using the sulphur hexafluoride (SF_6_) tracer gas technique [24,25,26], whereby each of the 24 animals from the new and old lines (the 12 heaviest animals from the new line and the old line) were assessed daily for 24 h for 5 consecutive days in April 2015. Animals from the cross line were not evaluated (it was expected to obtain an intermediary result and the SF_6_ technique is both costly and laborious). As such, this study aimed to quantify only the emissions of the new and old lines. The steers were fitted with halters and sampling canisters for gas collection 15 days before sampling in order to allow the animals to adapt to the devices, and thereby facilitate handling during the sampling period. Permeation tubes filled with known quantities of SF_6_ and release rates (1431 ± 59 ng/min) were administered orally to each of the 24 animals, 15 days prior to sampling, in order to allow the flow of tracer gas to stabilize in the rumen.

The animals were equipped with gas collection halters attached to pre-evacuated polyvinyl chloride (PVC) sampling canisters, made to permit 50% filling in 24 h. The collections started at 7:00 a.m., with the animals brought in from their pasture strip to the handling center in order to facilitate the exchange of equipment. The pressures of the sampling canisters were measured immediately after completing the 24-h collection period to evaluate their quality. If the final pressure was below that expected, this could mean that the sampling line was blocked or disconnected, while greater expected pressure could mean that leakage occurred at some point in the system. In the case of either of these situations, the halter and sampling canisters were replaced. After exchanging the collection containers, the animals were returned to their original pasture strip.

Daily samples were also collected to determine the concentration of CH_4_ and SF_6_ in the environment. The pressures, dates, and times of removal of the containers in both cases were noted for control.

### 2.6. Feedlot Phase: Diet and Animals Measurements

The feedlot finishing phase lasted for 105 days and occurred between June and September 2015, when the animals reached average weights of 354 ± 41 kg. The feedlot consisted of 4 collective pens of 396 m^2^, each equipped with drinking water, methane sampler (GreenFeed), and 2 automated troughs (GrowSafe Systems Ltd., Airdrie, Alberta, Canada), where they were offered water and feed ad libitum. The animals were allocated, respecting the same order as the 4 pasture areas (Table 1). The duration of the adaptation period was 20 days, with the animals receiving only corn silage in the first 10 days, 50% of concentrate with corn silage during the next 10 days, and 100% concentrate at the end of this period. The dietary composition is described in Table 2.

The experimental diet was offered twice daily at 8:00 a.m. and 2:00 p.m. Samples of the complete diet were collected on a weekly basis to determine the DM of the diet, using an oven at 65 °C for a period of 72 h. To measure the intake, the steers were fitted with a half-duplex electronic identification (EID) ear tag (Allflex Brazil, Joinville, SC, Brazil), and each automated trough was equipped with an antenna to detect animal presence, load cells to measure the feed amount, and a grid with adjustable vertical bars to allow only one animal at a time. The acquisition software continuously recorded the feed disappearance data and the presence of an animal at the trough when the EID transversed the vertical plane of the grid. The sum of the amounts of each visit over a 24-h period defined the daily feed intake [27].

Animals were weighed at the beginning and end of the feedlot period after a fasting (16 h) period, and at 28-day intervals during this period without fasting for monitoring of liveweight gain. The level of carcass fat of the animals was monitored in vivo using the ultrasound technique, with the images provided by an Aquila model Pie Medical appliance, equipped with a 3.5 MHz, 18 cm linear transducer, and using vegetable oil as the coupling agent. All measurements were taken between the 12th and 13th ribs on the *Longissimus thoracis* muscle, following the methodology of Herring et al. [28]. ADG, feed conversion (FC), and feed efficiency (FE) were then determined based on the measurements of liveweight gain and dry matter intake in feedlot.

### 2.7. Methane Emissions Sampling in the Feedlot

To determine enteric methane emissions during the finishing stage, four units of GreenFeed (GF) (C-lock Inc., Rapid City, SD, USA) automated system were used in the experimental feedlot area, with the equipment installed on the sides of the barns occupied by animals of the old and new lines. During the first seven days, access to the device remained unrestricted in order to encourage the animals to visit it. Once each animal, belonging to each plot, had been identified by the system, the access to the device was limited to restrict access to one animal at a time. The equipment was programmed using software provided by C-Lock Inc. to provide a maximum of six rotations of the feed dosing cup, with approximately 55 g per rotation, and intervals of 45 s between rotations, permitting a maximum provision of 330 g of pellet per visit. A maximum of five visits per day were permitted, with a minimum interval of 4 h and 45 min required between visits. Thus, if an animal returned to the device in a period shorter than this interval, the pellets were not dispensed.

The pellets used were commercial feed pellets (Presence do Campo, InVivo Nutrição e Saúde Animal, Paulínia, São Paulo, Brazil), chosen for their high fiber content and guaranteed content levels of 13% moisture, 14% crude protein, 3% ether extract, 14% mineral matter, 20% crude fiber, 1.2% calcium, and 0.6% phosphorus. Operation of the device was triggered by animals inserting their heads into the trough, and a radio frequency identification sensor (RFID) identified the chip fixed to the ear of the animal. Gas sampling was only initiated when the animal’s head was in the correct position, as identified by an infrared sensor, resulting in the distribution of pellets to attract and encourage the animal to keep its head in the correct position. This factor, alongside wind speed and direction, determined the success of the reading since the position of the animal’s head and the wind may have varied, but only data with uninterrupted measurements were considered valid for statistical analysis. Periodical cleaning and calibration of the equipment was carried out to guarantee the quality of gas measurements.

### 2.8. Sample Analysis for Methane Emissions

For chromatography analysis, the sampling canisters were pressurized with nitrogen (N_2_) to approximately 10% above atmospheric pressure, with the analysis conducted using a Shimadzu model GC-2014 chromatograph (Shimadzu Corporation, Kyoto, Japan) following the method described by Johnson and Johnson [29]. Calibration curves were established using standard gas certified by the company “White Martins” (São Carlos, São Paulo, Brazil) with CH_4_ concentrations in ppm (4.85% ± 5%, 9.96% ± 1.65%, and 19.1% ± 3.44%), and SF_6_ concentrations in ppt (34 ± 9.0, 91.0 ± 9.0 and 978.0 ± 98.0), in accordance with Johnson et al. [30].

The CH_4_ emissions were calculated using the CH_4_:SF_6_ ratio in the sampling canister, with each of the gases corrected for background concentrations, together with the predetermined permeation rate of the SF_6_ capsules. In the following equation, M indicates the sample measured from the animal and BG indicates the background concentration:

(2) $${\mathrm{RCH}}_{4}\;={\;\mathrm{RSF}}_{6}\;\times \;\frac{{\left(\mathrm{CH}4\right)}_{M}\;-\;{\left(\mathrm{CH}4\right)}_{\mathrm{BG}}}{{\left(\mathrm{SF}6\right)}_{M}\;-\;{\left(\mathrm{SF}6\right)}_{\mathrm{BG}}}\;\times \;\left(\frac{{\mathrm{MW}}_{\mathrm{CH}4}}{{\mathrm{MW}}_{\mathrm{SF}6}}\right)\;\times \;1000$$

Furthermore, RCH_4_ (g/d) is the ruminal CH_4_ emission rate calculated in g/d; RSF_6_ depicts the release rate measured from the SF_6_ capsule (mg/d); MWCH_4_ is the molecular weight of CH_4_ (16) and MWSF_6_ is the molecular weight of SF_6_ (146). The CH_4_ concentrations are expressed in ppm and SF_6_ concentrations in ppt. The factor 1000 converts the units so that RCH_4_ is expressed in g/d.

The flow of enteric methane emitted by the animal during the finishing stage was calculated, considering the methane concentration of the sample and the volume of air displaced by the extractor fan. Real-time communication between the GF units and the software provided by C-Lock Inc. on the internet was possible via a Wi-Fi network. Methane emission was expressed in the following different units: grams per day (g/day), kilograms of methane per kilogram of dry matter intake (kg/kg DMI), kilograms per kg of average daily gain (kg/kg ADG), grams per kg of liveweight (g/kg LW), and methane yield (YM%) as the percentage of gross energy lost in the form of CH_4_ (as proposed by Intergovernmental Panel on Climate Change-IPCC [31]). To calculate YM (%), the daily emission (CH_4_ g/d) transformed into unit kg (CH_4_ (g/d)/1000), was divided by the amount of energy of 1 kg of CH_4_ (55.6 MJ/kg). The value obtained was divided by the gross energy ingested (DMI/GE of 1 kg of the diet = 18.4 MJ/kg DM) and multiplied by 100; thus, obtaining the value of YM in percentage. For this calculation, the gross energy content of the diet (MJ/kg MS) was determined using a calorimeter pump (IKA C 200, Staufen, Germany); sub-samples were collected from the samples used to determine dry matter, and ground to 2 mm, before being shaped into pellets and burned inside the pump, according to AOAC recommendations [32].

### 2.9. Carcass Data Collection

The animals were slaughtered at the end of the feedlot phase, when they happened to have an average of 488 ± 48 kg of body weight and 4 ± 1.5 mm subcutaneous fat thickness, as measured by ultrasonography. The slaughter was carried out in accordance with Brazilian government inspection procedures.

After 16 h of fasting (water and feed), the animals were weighed to obtain the shrunk body weight (SWB). At slaughter, the animals were stunned with a captive bolt gun and then exsanguinated through jugular section. Carcasses were weighed before being stored in a cold chamber for 24 h, to obtain the hot carcass weight (HCW). After this interval, a sample of the *Longissimus thoracis* muscle was taken between the 12th and 13th ribs of the left half carcass for real rib eye area (REA) and fat thickness (FT) measurements, using tracing paper and reticulated grid (cm^2^), and a millimeter ruler, respectively. The weights of the main cuts hind quarter (HQ), front quarter (FQ), and spare ribs (SR) were also taken. The carcass dressing percentage (CD) were calculated as the ratio between the HCW and the SBW.

### 2.10. Statistical Analyses

Data were analyzed using the Statistical Analysis System 9.3 package (SAS Inst. Inc., Cary, NC, USA). The experimental unit was the individual animal for the analyzed variables. The presence of discrepant information (outliers) and normality of residuals (Shapiro–Wilk) were tested using the PROC UNIVARIATE procedure. Performance data (initial LW, final LW, ADG, DMI, FC, FE) and data relating to CH_4_ emissions (g/day; kg/year; kg/kg DMI; kg/kg ADG; g/kg LW; YM (%)) were analyzed using the PROC MIXED procedure for mixed models. Fifteen different covariance structures were tested, and the best-fitting statistical model was selected [33] based on the lowest value of Corrected Akaike Information Criterion (AICC). The model included the effect of treatment (line), period (pasture and feedlot) as a fixed effect, and random of block (area repetition).

The slaughter (HQ; FQ; SR; FT; REA) were analyzed using the same procedure for mixed models, but when the normality of the residuals was not met, the Poisson distribution—a method available in Proc Glimmix, a component of SAS—was also tested. The Tukey’s test was used to compare the treatments for all variables, considering a level of significance of 5%.

## 3. Results

The results obtained for the performance variables during the pasture rearing period are described in Table 3. The lines did not differ in initial LW, final LW, or DMI (*p >* 0.05), averaging 280.0 ± 32.6, 374.9 ± 42.9, and 6.3 ± 1.5 kg, respectively. The line of the animals had a significant effect on ADG (*p* < 0.001); the animals from the new line presented higher averages than the old line, while the animals belonging to the cross line did not differ from those of the new lines. 

The results for enteric methane emission variables corresponding to the pasture rearing period are described in Table 4. The line of the animals had no effect on methane emissions (*p* < 0.05) for the different units, averaging 203.4 ± 51.9 g/d, 0.331 ± 0.2 kg/kg ADG, and 0.560 ± 0.1 g/kg LW.

In the 105-day feedlot finishing period, the line used had an effect on the variables of ADG, daily DMI (*p* < 0.05), and FC (*p* < 0.01) (Table 5). The new line was superior (*p* < 0.05) in terms of ADG compared to the old line, while the cross line did not differ from the other two lines in relation to these variables. The highest daily DMI were observed in animals belonging to the new and cross lines in relation to the old line. The cross line presented a higher (*p* < 0.05) average FC compared to new and old lines.

Methane emissions from the feedlot finishing phase are described in Table 6. The line of the animals had an effect (*p* < 0.05) on CH_4_ emissions expressed in grams per day (g/day). Animals belonging to the new line emitted more methane (*p* < 0.05) than the old line when this variable was expressed in kilograms per year and grams per day. There were no differences in the methane emissions per DMI, ADG, LW, and YM, averaging, 0.017 ± 0.004 kg/kg DMI, 0.137 ± 0.1 kg/kg ADG, 0.375 ± 0.1 g/kg LW, and 5.13 ± 1.3%, respectively.

Table 7 shows the averages for CY, HQ, FQ, SR, FT, and REA. The line had a significant effect (*p* < 0.05) on the dressing of the animals given that FT was greater in the carcasses from the new line than the old line, while the FT measured from the cross line carcasses did not differ (*p >* 0.05) from the carcasses of the new and old lines. The genetic line did not affect any of the other variables measured on the carcass, although SR tended (*p* = 0.058) to be lower in the old line compared with the other two lines.

## 4. Discussion

The superior daily LW gain displayed by the new line during the rearing period was a result of the use of improved bulls to form this line, since feed intake did not differ between the animals of the two lines, as was also the case in relation to the cross line. According to Alencar [34], the Canchim breed’s performance characteristics generally show medium to high values for heritability, indicating that selection results in good genetic progress. This was backed-up by the superior performance of the new line in this study. Animal performance is an important aspect when the purpose of cattle rearing is meat production. Usually, the evolution of the animals is evaluated based on liveweight gain, which can be measured at different stages of the animal’s life (breeding, post-weaning, and finishing).

In a study of Canchim cattle in an exclusive grazing regime, Vieira [35] observed an ADG of 0.375 kg/day, lower than the figure obtained in this study for the new line (0.692 kg/day), old line (0.620 kg/day), and their cross (0.547 kg/day), grazing a pasture with high nutritional value and high availability. Vieira’s [35] study was one of the pioneering studies in relation to the performance of the Canchim breed, where genetic progress in the breed had previously been based solely on observations of the breeding of the different combinations of breeds, and not on the result of an already established selection process, without any perspectives for formation of a new line. Alencar et al. [36], comparing Canchim and Nellore cattle breeds of both sexes under a grazing regime, found differences in ADG and identified higher gains for Canchim males in relation to the other categories. Goulart et al. [37], working with Nellore and crossbreeds (Nellore, Canchim × Nellore, Angus × Nellore, and Simental × Nellore) reared on pasture, also observed different ADG (0.210, 0.290, 0.300, and 0.330 g/day, respectively), where the Nellore group presented lower ADG in relation to the three crossed groups, whilst for the variables FE and FC, significant differences were not found.

In the feedlot finishing phase, the highest ADG was presented by the new line, which can be explained by their higher feed intake and lower value for FC. The old line, in spite of its FC being equivalent to that of the new line, did not obtain similar ADG due to lower DMI. The cross line animals, despite having higher DMI than the old line, did not gain more liveweight because their FC was worse than the old line. Fernandes et al. [38], working in feedlots with Canchim animals of different sexual condition (castrated/not castrated males and females), observed significantly different ADG, DMI, and FC. Differences in these same characteristics were also observed by Rubiano et al. [39], who studied Nellore, Canchim, and the crossbred between these breeds in feedlots. Increased ADG is expected when there is higher DMI, since these characteristics are positively correlated [40]. The effect of the Charolais breed on performance was also confirmed by Crews et al. [41], who studied genetic parameters between growth and carcass traits of Charolais animals and stated that the heritability of these traits is moderate to high in this breed, indicating that selection can be used to improve both growth patterns and the conformation of the carcass.

The Canchim breed displays additive genetic variation in relation to LW gain, with improvements in this characteristic as time progresses, as described by Chaves et al. [42], and this was also confirmed by the results of the animals of the new line in this study. Barichello [43] also stated that the genetic selection process applied to the Canchim breed has been providing progress in LW and slaughter conformation characteristics. In the classic study reported by Koch et al. [44], they describe how selection for LW gain is effective and leads to improved FE, in addition to an increase in feed intake. These effects were observed in this study in the new line (formed from improved 1990s Charolais bulls with proven genetic merit) when compared to the old line (closed line since 1960). 

Regarding enteric methane emissions in the pasture phase, different studies have compared the emissions obtained using the SF_6_ tracer gas technique with results obtained from the same animals in respiration chambers, and observed average variations in emissions of 7.5% either way [45]. As such, the technique provides greater variability in results causing the need to increase the number of animals necessary to identify treatment differences [46]. Twelve animals per treatment measured using the SF_6_ technique in this experiment is not a small number, but may not have constituted enough animals to detect differences in methane production between the new and old line. According to Mercadante et al. [47], there is no evidence that more efficient animals produce less enteric methane, even if they display lower feed consumption and performance equivalent to animals considered inefficient. Sakamoto et al. [48], evaluated emissions from Canchim cattle reared in different pasture systems (intensive, extensive, integrated crop-livestock, integrated crop-livestock-forest and silvopastoral) and did not find significant differences in CH_4_ emissions in grams per day.

The higher emissions of enteric methane from the new line animals in the feedlot can be related to the higher DMI, since there is a directly proportional relationship between increased consumption and increased methane production [49,50]. This finding was also reported by Moss et al. [51], who highlighted the fact that methane production is linked to DMI, but this variable should only be used with single DM sources or diets, as in this study. It is also important to note that other authors attest to a strong correlation (r > 0.60) between methane production and DMI [31,40]. Hegarty et al. [52], working in feedlots with Angus lines selected for low or high residual fed intake (RFI), observed significant differences in relation to DMI and daily methane production; the animals that presented higher DMI emitted greater quantities of enteric methane. However, the authors did not observe differences in ADG and methane production expressed in relation to ADG or DMI, as was also the case in this study. Genetic selection for lower methane production can produce undesirable effects on animal productivity, as this characteristic has a strong correlation (0.79 to 0.86) with growth characteristics [53]. In this study, despite the old line emitting less methane, it also demonstrated lower ADG and higher FC.

Although a higher emission is expected as the animals grow [54], in this experiment we observed higher emissions in the pasture period. The difference between pasture and feedlot periods can be explained by the different methods used for measuring the CH_4_. According to Hammond et al. [55], the emissions can be higher using SF_6_ technique rather than GF automated system, but there is a significant correlation (0.60) between the two methods. Another factor is the different diets in the two periods; feeding grain-based diets can lower enteric CH_4_ emission when compared with forage-based diets [29], which is in agreement with the present experiment, since the emissions decreased when the animals went from the pasture to feedlot. The gross energy content in the diet also supports this statement, as the animals had more than twice the energy available in the feedlot (8.7 ± 0.1 vs. 18.4 ± 0.1 MJ/kg DM). In general, the average methane emissions determined in this experiment (67 kg/year) are consistent with estimates made using the IPCC’s Tier 2 methodology (64 kg/year) [56], slightly above the Brazilian national average, estimated at 60 kg/year [57,58].

The greater FT in steers of the new line in this study may be linked to the higher ADG of the animals during the rearing period on pasture and feedlot finishing. According to Di Marco et al. [59], breed, dietary history, and increase of liveweight gain are characteristics that determine the amount of subcutaneous fat deposited. A few studies show results related to fat deposition: Fernandes et al. [38], evaluating the performance and carcass characteristics of castrated or non-castrated males, plus females of the Canchim breed, detected similar deposition of subcutaneous fat between the different categories evaluated. The Canchim breed displays delayed fat deposition, with this feature becoming more significant as the animal nears adult liveweight and, according to Alencar et al. [60], the use of the Charolais breed provides a higher growth rate and larger size, in addition to other features, with subsequent deposition of fat at a later stage.

Despite the differences in FT observed between the new and old lines, all met the minimum of 3 mm required by slaughterhouses. The FT has an important role in the process of transforming muscle into meat and is a determining factor in its quality since it acts as a thermal insulator to ensure that the temperature decrease occurs more slowly, protecting the carcass [61,62], and preventing the shortening of muscle fibers by the cooling action [63]. This change began in the early 1990s, where practically all beef came from lean male animals of 3 to 5 years of age [39]. Since then, the reduction in the age at slaughter, associated with the genetic potential of the animals, has been an important step towards producing meat with greater efficiency and of higher quality, satisfying the final consumer.

## 5. Conclusions

Reared on pasture, the steers of the new genetic line of the Canchim breed grow faster and emit the same amount of enteric methane as those of the old line. Additionally, when finished in feedlot, the steers of the new line grow faster than the steers from the old line, but emit a higher amount of methane per day. In the feedlot phase, the animals from the new and the crossed lines have a higher dry matter consumption than those of the old line, whilst the new and the old lines are more efficient in converting the feed consumed into liveweight, compared with the cross line steers. Moreover, better carcass dressing can be obtained by using the new lines of the Canchim breed, when reared on pasture and finished in feedlot. These results are evidence of the impact that the genetic improvement exerted on the Canchim composite breed have on animal performance and methane emissions.

## Figures and Tables

**Table 1:** Animal arrangement according their line in the pasture areas and feedlot pens, block initial liveweight (B-ILW), and line initial liveweight (L-ILW).

Pasture Area and Feedlot Pen						
Line	A	B	C	D	Total	L-ILW (kg)
OL	3	3	3	4	13	280.5
CL	3	4	4	2	13	286.8
NL	5	5	5	5	20	274.6
B-ILW (kg)	305.2	288.0	280.0	244.8	46	

**Table 2:** Percentage of ingredients and nutritional composition of the diet used in the feedlot based on dry matter (% DM).

Ingredients	Proportion of the Diet (% DM)
Corn silage	37.7
Corn Grain Milled	35.5
Soybean meal	12.5
Wheat bran	10.9
Calcitic limestone	1.1
Mineral Mixture ^A^	1.5
Urea	0.8
Sodic Monensin ^B^	0.03
**Nutritional Composition of the Diet (g/kg)**	
CP	152
TDN	721
NDF	322
EE	44
Ca	10
P	5.5

CP, crude protein; TDN, total digestible nutrients; NDF, neutral detergent fiber; EE, ether extract; Ca, calcium; P, phosphorus. ^A^ Guaranteed levels per kg of product: Calcium: 176 g; Iodine: 0.084 g; Phosphorus: 65 g; Manganese: 1 g; Zinc: 4.5 g; Cobalt: 0.06 g; Fluorine: 650 g; Copper: 1 g; Iron: 1.9 g; Selenium: 0.015 g; Magnesium: 12 g; Sulfur: 12 g; Sodium: 114 g; Zinc: 4.5 g; Cobalt: 0.06 g. ^B^ Monensin content in the product: 200 g/kg.

**Table 3:** Performance and efficiency of Canchim steers from different lines reared on pasture.

Variables	Treatments			s.e.m.	*p*-Value
	New	Crossed	Old		
ILW (kg)	275	287	282	5.75	0.7357
FLW (kg)	379	383	362	6.65	0.6433
ADG (kg/day)	0.692a	0.620ab	0.547b	0.04	0.0002
DMI (kg/day)	6.49	6.38	6.06	0.15	0.2717

Means followed by different lowercase letters on the same line differ from each other (*p* < 0.05), according to the Tukey test. s.e.m., standard error of the mean; ILW, initial weight; FLW, final weight; ADG, average daily gain; DMI, dry matter intake, of lines of the Canchim.

**Table 4:** Methane emission factors of Canchim steers from different lines reared on pasture.

CH_4_ Emissions	Treatments		s.e.m.	*p*-Value
	New	Old		
g/day	213	194	10.63	0.3835
kg/kg ADG	0.324	0.337	0.0194	0.7483
g/kg LW	0.562	0.557	0.0260	0.9304

Means followed by different lowercase letters on the same line differ from each other (*p* < 0.05) according to the Tukey test. s.e.m., standard error of the mean; ADG, average daily gain; LW, liveweight.

**Table 5:** Performance and efficiency of Canchim steers from different lines finished in feedlot.

Variables	Treatments			s.e.m.	*p*-Value
	New	Crossed	Old		
ILW (kg)	379	383	362	7.43	0.2607
FLW (kg)	524	517	485	7.86	0.2008
ADG (kg/day)	1.44a	1.32ab	1.23b	0.06	0.0144
DMI (kg/day)	10.25a	10.42a	9.11b	0.20	0.0289
FC (kg DM/kg ADG)	6.92b	9.10a	7.46b	0.29	<0.0001
FE (%)	14.5	13.1	14.0	0.57	0.1544

Means followed by different lowercase letters on the same line differ from each other (*p* < 0.05) according to the Tukey test. s.e.m., standard error of the mean; ILW, initial weight; FLW, final weight; ADG, average daily gain; DMI, dry matter intake; FC, feed conversion; FE, feed efficiency.

**Table 6:** Methane emission factors of Canchim steers from different lines finished in feedlot.

CH_4_ Emissions	Treatments		s.e.m.	*p*-Value
	New	Old		
g/day	178a	156b	7.39	0.0155
kg/kg DMI	0.017	0.016	0.0007	0.2592
kg/kg ADG	0.149	0.124	0.011	0.0578
g/kg LW	0.379	0.371	0.0179	0.7235
YM (%)	5.19	5.07	0.25	0.6901

Means followed by different lowercase letters on the same line differ from each other (*p* < 0.05) according to the Tukey test. s.e.m., standard error of the mean; DMI, dry matter intake; ADG, average daily gain; LW, liveweight; YM (%), percentage of gross energy in feed converted to methane.

**Table 7:** Carcass traits and main cut weights of Canchim steers from different lines.

Variables	Treatments			s.e.m.	*p*-Value
	New	Crossed	Old		
CD (%)	53.1	53.4	53.2	0.31	0.6902
HQ (kg)	130	129	121	3.40	0.1268
FQ (kg)	98.9	98.7	92.4	2.57	0.1565
SR (kg)	34.8	33.9	30.7	1.22	0.0581
FT (mm)	4.4a	3.8ab	3.2b	0.40	0.0131
REA (cm^2^)	76	71	73	2.07	0.2857

Means followed by different lowercase letters on the same line differ from each other (*p* < 0.05) according to the Tukey test. s.e.m., standard error of the mean; CD, carcass dressing; HQ, hind quarter; FQ, front quarter; SR, spare ribs; FT, subcutaneous fat thickness; REA, ribeye area.
